# *P. falciparum In Vitro* Killing Rates Allow to Discriminate between Different Antimalarial Mode-of-Action

**DOI:** 10.1371/journal.pone.0030949

**Published:** 2012-02-23

**Authors:** Laura M. Sanz, Benigno Crespo, Cristina De-Cózar, Xavier C. Ding, Jose L. Llergo, Jeremy N. Burrows, Jose F. García-Bustos, Francisco-Javier Gamo

**Affiliations:** 1 Tres Cantos Medicine Development Campus, Diseases of the Developing World, GlaxoSmithKline, Tres Cantos, Madrid, Spain; 2 Medicines for Malaria Venture, Geneva, Switzerland; Bernhard Nocht Institute for Tropical Medicine, Germany

## Abstract

Chemotherapy is still the cornerstone for malaria control. Developing drugs against *Plasmodium* parasites and monitoring their efficacy requires methods to accurately determine the parasite killing rate in response to treatment. Commonly used techniques essentially measure metabolic activity as a proxy for parasite viability. However, these approaches are susceptible to artefacts, as viability and metabolism are two parameters that are coupled during the parasite life cycle but can be differentially affected in response to drug actions. Moreover, traditional techniques do not allow to measure the speed-of-action of compounds on parasite viability, which is an essential efficacy determinant. We present here a comprehensive methodology to measure *in vitro* the direct effect of antimalarial compounds over the parasite viability, which is based on limiting serial dilution of treated parasites and re-growth monitoring. This methodology allows to precisely determine the killing rate of antimalarial compounds, which can be quantified by the parasite reduction ratio and parasite clearance time, which are key mode-of-action parameters. Importantly, we demonstrate that this technique readily permits to determine compound killing activities that might be otherwise missed by traditional, metabolism-based techniques. The analysis of a large set of antimalarial drugs reveals that this viability-based assay allows to discriminate compounds based on their antimalarial mode-of-action. This approach has been adapted to perform medium throughput screening, facilitating the identification of fast-acting antimalarial compounds, which are crucially needed for the control and possibly the eradication of malaria.

## Introduction

With more than 220 million cases and 781,000 deaths reported in 2009, the heavy malaria burden demands urgent solutions [1]. Due to renewed efforts and investments, the number of fatalities has been, however, constantly decreasing since 2004 and led the malaria community to re-embark on the long-term goal of eradicating this parasitic disease [2]. This ambitious endeavor will require innovative tools to be widely available, including innovative antimalarial drugs, to ensure the best possible control of this disease and, hopefully, its eradication [3]. New chemotypes and new modes-of-action are needed not only to address eradication-specific objectives but also to replace drugs loosing efficacy due to the advent of resistant parasites [3]. A critical example of the latter comes from artemisinin derivatives, which are used as combination for first-line therapy in the vast majority of endemic countries. Resistance to artemisinins seems to be emerging in South-East Asia, as characterized by *in vivo* delayed parasite clearance times (PCTs) [4]. Not only genetic markers to monitor this emerging resistance are missing, but so are potential replacement drugs [5], [6]. This situation underlines our current need for new antimalarial drugs, which should ideally have the fastest possible speed-of-action, in order to maximize therapeutic efficacy and minimize the in-patient opportunity window for resistant parasite selection and dissemination.

A number of *in vitro* techniques have been developed to investigate the effect of drug treatment on asexual parasite survival and growth, and are used for both drug development and resistance monitoring studies [7]. Standard techniques use different approaches, but they all have in common to measure the growth inhibition of intraerythrocytic parasites after their exposure to antimalarial compounds for a variable amount of time, generally between 24 and 96 hours. This measure is obtained through either counting parasites by microscopy, measuring the presence of nucleic acids, specific proteins or metabolites, or the incorporation of precursor thereof, or measuring the activity of parasite specific enzymes. The most simple assay is the “WHO micro-test”, which was established in 1978 and is based on the measurement of schizont maturation by microscopic examination of thick blood films after 24 or 48 hours of drug treatment [8]. This assay requires only simple material but is very labor intensive and does not permit to investigate large set of compounds simultaneously. These limitations prompted for the development of alternative methods such as the isotope-based assays. These rely on the incorporation of radio-labeled hypoxanthine or other metabolic precursor as a measure of parasite growth [9], [10]. In contrast to the “WHO micro-test”, drug treatment can be extended for up to 96 hours and the onset and duration of the labeled precursor incorporation can be adapted, improving measurement flexibility and reproducibility. Importantly, these assays can be, at least partially, automated and are therefore amenable to screening large set of compounds. Additional assays, which avoid the use of radio-labeled material and of relatively expensive scintillation counters have been more recently developed and are based on the measurement of enzyme presence or activity. The parasite lactate dehydrogenase (pLDH) enzyme is involved in the glycolysis pathway and can be used as a marker for the parasite presence. Colorimetric as well as immunodetection-based assays have been developed to measure this protein both, in laboratory and field conditions [11], [12]. The histidine-rich protein 2 (HRP2) is another parasite specific protein which can be measured by immunodetection, providing a precise estimation of the parasite growth rate [13]. Taking advantage of the fact that erythrocytes are devoid of DNA material, alternative methods have been developed to quantify the parasite nucleic acids, using fluorescent dyes such as SYBR Green or DAPI [14], [15]. Finally, flow cytometry has also been used to measure light depolarization by heamozoin or DNA content, providing new methods to evaluate drug potency [16], [17]. These assays differ in sensitivity, application, and cost, but they all have in common to measure parasite viability in an indirect manner. Relying on proxies to measure viability can have a significant impact on the results obtained with these different assays and might render their interpretation particularly challenging or erroneous [18]. For instance, viable but metabolically inactive parasites will be measured as dead. This scenario is naturally occurring during the parasite life cycle, such as in mature gametocytes or *P. vivax* hypnozoites, which are arrested in G_0_ [19], [20]. The converse is also true; parasites committed to death can still display metabolic activities or steady levels of marker expression and be classified as viable by the aforementioned assays. Such a situation can be induced by drug treatment itself, as exemplified by the choline analog T3/SAR97276 [18]. This compound efficiently kills *P. falciparum* after 2 hours of exposure, however pLDH- and HRP2-based assays fail to detect these effects, suggesting that ongoing marker accumulation is masking it. Delayed parasite death, as observed with different antibiotics, including azithromycin and clindamycin, will also lead to erroneous viability assumptions from assay based on microscopy observations or metabolic activities [21], [22]. It is also possible that drug treatment might directly affect the metabolic pathways measured, without altering parasites viability, which would also lead to erroneous measurements. These different situations show that metabolic activity and viability are two parameters which can be uncoupled either naturally or in response to drug treatment and that relying on the first to measure the latter can be a possible source of artefacts. Moreover these assays do not permit to directly measure the speed-of-action of antimalarial compounds on parasite viability, which is nevertheless a crucial parameter for anti-infective activities and clinical efficacy.

The net effect of antimalarial compounds is primarily mediated by their ability to kill parasites and decrease parasitemia so that patient cure can be achieved and parasites recrudescence prevented. Killing rates can be estimated *in vivo* by the parasite reduction ratio (PRR), which is the ratio between parasitemia at the onset of drug treatment and 48 hours later, corresponding to one asexual parasite life cycle [23]. The reduction of circulating parasitized erythrocytes generally follows a log-linear curve and this measure can be used as a predictive therapeutic index. PRR values vary greatly, with fast-killing drugs, such as artemisinin, achieving up to 99.99% decrease in parasitemia during one life cycle, resulting in an *in vivo* apparent PRR in the order of magnitude of 10^3^–10^4^, while slower ones, such as clindamycin, might exert a PRR of only 10 [24], [25]. Beyond the killing rate itself, the effect of antimalarial drugs also depends on the type of growth inhibition involved. Cytotoxic drugs, by actively killing parasites, generally display a high level of efficacy while cytostatic drugs, such as atovaquone [26], might not completely eliminate circulating parasites and lead to treatment failure and resistance selection. As illustrated above, metabolism-based assays are generally poorly suited tools to discriminate between cytotoxic and cytostatic activities.

We present here an extensive set of data using an *in vitro* methodology developed to circumvent the limitations of classical metabolism-based assays in measuring anti-malarial activities and compound killing rates. This approach is based on the direct measurement of intraerytrocytic *P. falciparum* viability in response to drug treatment over time. *In vitro* PRR as well as PCT values can be derived from these measures. Moreover, drug lag phases, that is the time required for a drug to achieve its maximal killing effect, can be precisely identified and timed. The detailed characterization of several antimalarial drugs from various chemical classes is presented, providing both a validation of our methodology as well as benchmark data for further studies on compounds in development.

## Results

### Assessing *P. falciparum* viability *in vitro*

**Figure 1:**
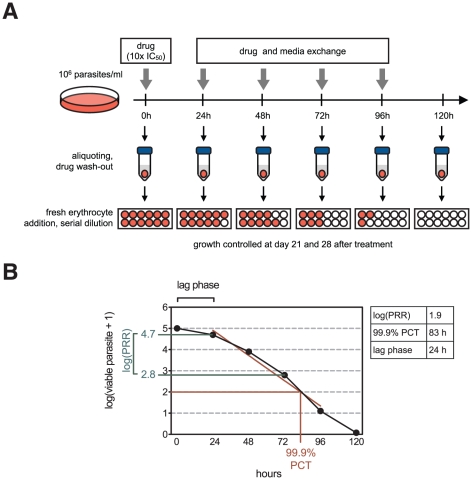
Schematic representation of the *in vitro* PRR assay. **A**. Intraerythrocytic *P. falciparum* cultured at 0.5% parasitemia and 2% hematocrit is treated with drugs. The medium is exchanged and the drug replenished every 24 hours. Aliquots corresponding to 10^5^ parasites are taken out at defined time points, washed, and free-drug parasites cultured with fresh erythrocytes under limiting serial dilution conditions (see Material and Methods). Parasite growth is subsequently monitored after 21 days and confirmed after 28 days, allowing to calculate the initial number of viable parasite in the aliquot. **B**. Parasite viability measurement allows in turn to determine the drug lag phase (i.e. time needed to reach the maximal rate of killing), PRR over one life cycle, and 99.9% PCT (i.e. the time needed to decrease the number of viable parasites by 3 –log units). The data presented in this panel are for illustration purpose only. Axe Y shows log (viable parasites +1) to allow representation of logarithms when counting of number of viable parasites is equal to zero.

A comprehensive methodology has been developed to directly measure the net effect of chemical compounds on the viability of intraerythrocytic asexual forms of *P. falciparum* parasites (Figure 1A). The principle of the method is the following. An initial inoculum of 10^6^ intraerythrocytic parasites per milliliter, as determined by microscopy, is established at 0.5% parasitemia and 2% hematocrit. These conditions are exactly identical to the ones used for standard IC_50_ determination using a tritiated [^3^H]-hypoxanthine incorporation assay [9]. To avoid multiple infections, original cultures are incubated under shaking conditions. Resulting cultures present less than 1% erythrocytes with multiple infections. Once drug treatment is initiated, the effect of the inhibitor on parasite viability is monitored by taking out an aliquot corresponding to 10^5^ parasites of the initial population every 24 hours, washing it thoroughly, adding fresh erythrocytes and performing serial dilutions in a microtiter plate. The number of parasites in the initial inoculum is calculated by performing the same actions with an aliquot of the culture before starting treatment. The limiting serial dilution culture is maintained for up to 28 days and the presence of growing parasites is terminally determined in each well by using any standard technique able to detect parasite growth, such as pLDH detection or [^3^H]-hypoxanthine incorporation. The number of viable parasites initially present in the aliquot, that is the number of parasites able to recrudesce upon removal of the drug, can then be back-calculated based on the most diluted well able to render growth. By repeating this procedure for multiple time points, here every 24 hours for up to 120 hours, it is possible to obtain a direct measurement of parasite viability over time in response to drug treatment. *In vitro* PRR is calculated as the decrease in viable parasites over 48 hours, that is one parasite life cycle, and is a direct measurement of the killing rate for the compound investigated (Figure 1B). Hence, a compound leaving 10^3^ parasites alive out of 10^5^ after 48 hours of treatment has a PRR of 10^2^ (10^5^/10^3^) or log_10_(PRR) of 2, hereafter referred to as log(PRR). A “lag phase” is considered to occur for as long as drug treatment does not produced the maximal rate of killing, and this period of time is excluded for PRR calculation. In a practical way, 0–24 or 0–48 hours stretches are considered part of the lag phase when estimated reduction of parasite viability over 48 hours, (extrapolated from 0–24 hours in the first case), is more than one order of magnitude below the calculated PRR using the linear stretch of the profile. It is important to note that a lag phase is not observed in all profiles. 99.9% PCT, that is the time needed to clear 99.9% of the initial parasite population, is determined using a regression calculated on the log-linear phase of the parasite reduction and takes the lag phase into account (Figure 1B). In summary, measuring parasite viability over time in response to drug treatment allows to determine key *in vitro* parameters of the compound killing rate such as lag phase, PRR and 99.9% PCT values.

Several steps have been undertaken to validate this viability-based approach. First, aliquots of a known number of parasites, as determined by microscopy, have been used to evaluate the precision with which the serial limiting dilution technique allows to calculate the number of viable parasites. 1/2, 1/3, and 1/10 dilutions of parasite aliquots have been performed (Figure S1). Every dilution factor tested could accurately be used to back calculate the initial number of parasite population, with values ranging from 10^4.7^ to 10^5.2^ for aliquots of 10^5^ parasites. 1/3 dilutions have been further used for the experiments presented in this study. Because drugs vary in potency and in order to compare their effects, treatments were performed at concentrations based on the drug-specific 50% inhibitory concentration (IC_50_), which is the concentration required to inhibit the growth of a parasite population by 50%. IC_50_ values for the drugs investigated in the present study have been determined in house using a classical hypoxanthine incorporation based assay (Table S1) and, if not otherwise stated, concentrations corresponding to 10 fold IC_50_ have been used for the experiments reported here. Moreover, to ascertain a constant level of parasite exposure to drugs, the latter are replenished every 24 hours by exchanging the culture media, which is especially important for rapidly degrading compounds, such as artemisinin [27]. Artemisinin treatment led to a rapid decrease in viable parasites, from 10^5^ to virtually 0 in 48 hours (see below). However if the initial drug level is not kept relatively constant, by replacing the media every 24 hours, the number of viable parasite increases at 96 and 120 hours after drug addition (Figure S2). This suggests that rapid artemisinin degradation, to a level below the minimum inhibitory concentration, might permit surviving parasites to resume an active growth. Degradation of artemisinin in culture media has been confirmed by quality control analysis of the media (data not shown).

### Metabolism is not a good surrogate of parasite viability

To explore the drug antimalarial effects on metabolism and parasite viability, we have investigated compounds with very different antimalarial mechanism of action: atovaquone, azithromycin, and artemisinin. Atovaquone is an ubiquinone analog that disrupts electron transport chain function by targeting the cytochrome *bc*_1_ complex of *Plasmodium* spp. and is currently used as a treatment in combination with proguanil [28]. Azithromycin is a widely used antibiotic with demonstrated antimalarial activity but showing a delayed death phenotype on malaria parasites possibly through inhibition of the bacterial-like apicoplast translation machinery [21]. We compared the effect of these drugs on parasite hypoxanthine incorporation, as a measure of metabolic activity, and on the number of parasites alive after treatment, as described above.

Standard [^3^H]-hypoxanthine incorporation assay was used to construct dose response curves and calculate IC_50_ of 1 and 32 nM for atovaquone and artemisinin respectively (Table S1). In these conditions, azithromycin did not produce any reduction of [^3^H]-hypoxanthine incorporation when compared with the control, even at the highest concentration tested (10 µM). However, an IC_50_ of 0.27 µM, could be determined by extending the treatment time to 96 hours, confirming the time delayed phenotype described for this class of compounds. This IC_50_ value was used in the parasite viability assay.

**Figure 2:**
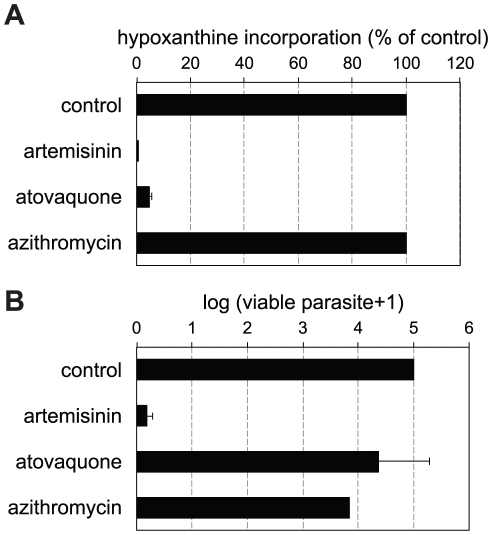
Comparison of metabolic and viability assays. **A**. *P. falciparum* radio-labeled hypoxanthine incorporations after 48 hours of drug treatment at concentrations corresponding to 10× IC_50_ with artemisinin, atovaquone, and azithromycin, reported as percentages of untreated controls. Data are averages of 12 repetitions from two independent experiments **B**. *P. falciparum* viability after 48 hours of drug treatment at concentrations corresponding to 10× IC_50_ with artemisinin, atovaquone, and azithromycin, reported as number of viable parasite, as determined by limiting serial dilutions. Data are averages of 4 independent experiments. In both panels, error bars represent the standard error of the mean (SEM).

The percentages of hypoxanthine incorporation observed after 48 hours at 10× IC_50_, relative to controls without drug treatment were negligible for artemisinin and atovaquone but 100% in the case of azithromycin (Figure 2A). Parasite viability was estimated under exactly the same conditions, that is after 48 hours of treatment at 10× IC_50_. Virtually no viable parasites were found after artemisinin treatment (ca. 5 log reduction in parasite load), in agreement with the absence of metabolic activity under these conditions (Figure 2B). However, in the case of atovaquone treated parasites, less than 1 log reduction in the number of parasites was observed despite the absence of metabolic activity. This suggests that these parasites are not committed to death and would be wrongly classified as such by metabolism-based assays. The response to azithromycin treatment displays a mirror image: treatment produces a decrease of more than 1 log in the parasite load despite fully metabolically active parasites are observed under these treatment conditions. These results illustrate the fact that metabolic activity levels do not always correlate with drug-induced death rate.

### Killing rate of classical antimalarial drugs

**Figure 3:**
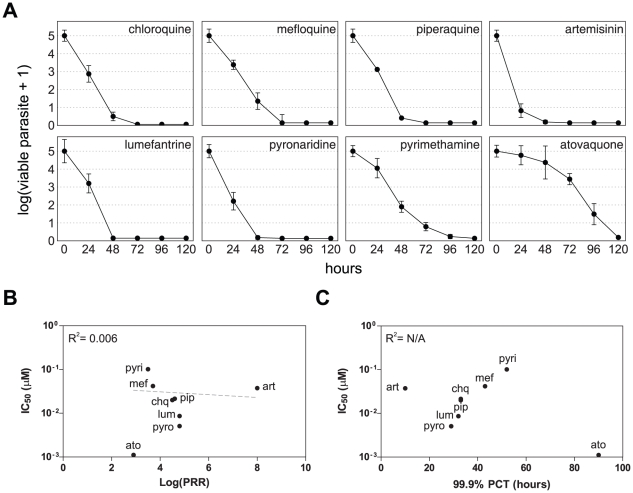
Parasite viability in response to various classical antimalarial drugs. **A.** *P. falciparum* viability time-course profiles for chloroquine (chq), mefloquine (mef), piperaquine (pip), artemisinin (art), lumefantrine (lum), pyronaridine (pyro), pyrimethamine (pyri), and atovaquone (ato). Error bars represent the SEM of at least 4 independent experiments. **B**. and **C**. Scatter plots of the compounds tested reporting the IC_50_ versus the log(PRR) and 99.9% PCT, respectively. The dotted line in panel B is a log linear regression, the slope thereof is not significantly different from zero (*p* = 0.48). Data of panel C do not converge enough to establish a regression line.

The killing rate parameters of an extended set of antimalarial drugs were determined. These include the quinoline-type chloroquine, mefloquine, and piperaquine, the amino-alcohols lumefantrine and pyronaridine, the dihydrofolate reductase (DHFR) inhibitor pyrimethamine, as well as atovaquone and artemisinin. Parasite viability time course profiles are presented in Figure 3A, lag phase, PRR and 99.9% PCT values are reported in Table 1. For all the compounds investigated, the number of viable parasites fell below 0.0001% of the starting population after 120 hours of treatment at the latest, indicating that the time-frame considered is appropriate to fully encompass the activity of these antimalarials. Two compounds displayed a significant lag phase: atovaquone for 48 hours and pyrimethamine for 24 hours and are also the slowest acting ones with log(PRR) and 99.9% PCT values of 2.9 and 90 hours for atovaquone and 3.5 and 52 hours for pyrimethamine. Artemisinin, on the other hand, is strikingly faster than any other compound in this set, with a reduction of parasite viability below 0.1% in less than 24 hours and a log(PRR) value higher than 8 (as extrapolated from the first 24 hours of treatment). It is followed, in decreasing speed-of-action order, by pyronaridine, lumefantrine, piperaquine, chloroquine, and mefloquine with PRR values of 4.8, 4.8, 4.6, 4.5 and 3.7, respectively. We next analyzed the relationship between IC_50_, log(PRR), and 99.9% PCT (Figure 3B–C). No significant correlation could be established between PRRs or PCTs and IC_50_, indicating the absence of a direct link between antimalarial potency and killing rate. Consequently, the speed-of-action of a compound cannot directly be predicted by its efficiency in blocking parasite hypoxanthine incorporation. These experiments show that antimalarial drugs display a wide range of killing rates, with 99.9% PCTs, ranging from less than 24 to 90 hours, as dictated by compound-specific lag phase and PRR, and independently of their potency.

**Table 1:** *In vitro* parasite reduction ratio and clearance time in response to classical antimalarial drugs.

	lag phase (h)	log(PRR)	99.9% PCT (h)
artemisinin	0	>8.0^a^	<24.0
pyronaridine	0	4.8	29
lumefantrine	0	4.8	32
piperaquine	0	4.6	33
chloroquine	0	4.5	32
mefloquine	0	3.7	43
pyrimethamine	24	3.5	55
atovaquone	48	2.9	90

a estimation based on the first 24 hours of treatment.

### Determination of the optimal dose for maximal rate of killing

**Figure 4:**
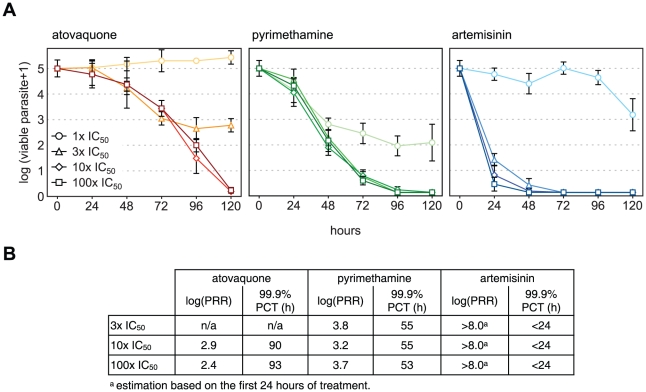
Parasite viability time-course in response to various concentrations of drugs. **A**. *P. falciparum* viability time-course profiles for atovaquone, pyrimethamine, and artemisinin at concentrations corresponding to 1×, 3×, 10×, and 100× their respective IC_50_. Error bars are SEM of at least 4 independent experiments. **B**. Values represented in panel A. No PRR or 99.9% PCT could be calculated for the 1× IC_50_ conditions.

Relatively large killing rate differences occurred with the different compounds tested. Considering that they all have been tested at 10× IC_50_, these results might reflect suboptimal dosing and, consequently, underestimated killing rate for some of the inhibitors. In order to test this hypothesis, *in* vitro PRR experiments were conducted with a representative subset of antimalarial drugs using concentrations corresponding to multiples of their respective IC_50_: 1×, 3×, 10×, and 100× (Figure 4). Atovaquone treatment at 1× IC_50_ displayed a plateau with a constant, or slightly increasing parasitemia, indicating that the rate of killing was compensated by the rate of growth in the treated culture. Increasing atovaquone concentration to 3× IC_50_ decreases the parasitemia level at which the plateau is reached. This indicates a net killing effect of the drug, as growth of the remaining parasites can compensate only partially the degree of killing, inducing an equilibrium at a parasitemia 2 logs below the starting inoculum. The treatment at 10× IC_50_ did permit to reach a virtually complete parasite clearance with a log(PRR) of 2.9 and a 99.9% PCT of 90 hours, while a further 10 fold increase of drug concentration did not significantly modify the killing rate profile observed, confirming that the maximal rate of killing was achieved at the previous concentration (10× IC_50_) (Figure 4). Pyrimethamine showed a similar pattern and, although at 1× IC_50_, it also failed to clear all the parasites within 120 hours, killing was higher than in the case of atovaquone 1× IC_50_ and similar to 3× IC_50_. This last concentration (3× IC_50_) appears however to be enough to reach the maximal killing rate of this compound, with log(PRR) and 99.9% PCT reaching values similar to the ones seen in response to 10× and 100× IC_50_ concentrations. For artemisinin, 1× IC_50_ is similarly not sufficient to clear the parasites within 120 hours. However a killing rate similar to that seen with 10× IC_50_ is achieved with as low as 3× IC_50_ and is not significantly different when the parasites are exposed to a dose corresponding to 100× IC_50_. This set of experiments shows that the maximal killing rate of these compounds is reached with doses corresponding to at most 10× IC_50_. This appears to be true for slow, medium, as well as fast killing compounds, as illustrated with atovaquone, pyrimethamine, and artemisinin, respectively. This observation is also true for additional compounds, including chloroquine and lumefantrine, which reached maximum killing rate at concentrations below 10× IC_50_ (Figure S3 and Table S2). Interestingly, in the case of chloroquine maximal rate of killing was observed at concentrations close to 1× IC_50_. For both, atovaquone and pyrimethamine, varying the compound concentration did not alter the lag phase duration, suggesting that this initial effect does not depend on the intensity of the parasite exposure to the drugs but rather on their intrinsic modes-of-action. Altogether, this suggests 10× IC_50_ to be an optimal dose to investigate compound speed-of-action at the highest rate of killing and to reflect true variations in compound ability to clear *P. falciparum* parasites.

### Compound rate of killing profile correlates with antimalarial mode-of-action

**Figure 5:**
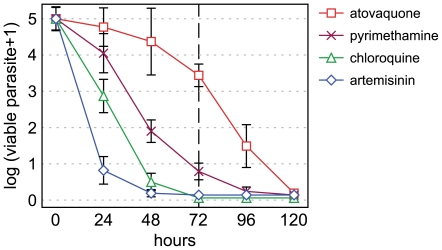
Classical antimalarial killing rate profiles. *P. falciparum* viability time-course profiles of four classical antimalarial drugs, illustrating the wide range of speed-of-action measured. Viable parasite are cleared in response to fast-acting (art and chq), but not slow-acting (pyri and ato) drugs after 72 hours of treatment.

**Figure 6:**
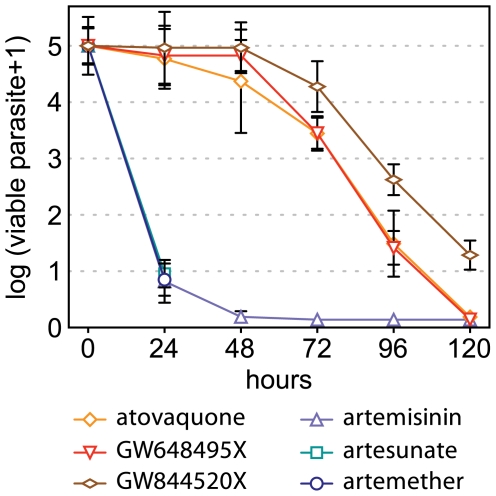
Parasite viability correlates with the mode-of-action of antimalarials. *P. falciparum* viability time-course profiles for artemisinin, atovaquone, GW648495, and GW844520 measured at 0, 24, 48, 72, 96, and 120 hours. Artemether and artesunate have been investigated at 24 hours only. Error bars are SEM of at least 4 independent experiments.

It is likely that, all things being equal, compound killing rates are primarily dictated by the antimalarial mode-of-action and that a specific profile could be representative for mechanism of killing (Figure 5). In order to substantiate this possibility, we next analyzed two sets of compounds sharing either the same pharmacophore or targeting the same pathway. The first set includes artemisinin and two derivatives artesunate and artemether, which all contain the endoperoxide bond required for their antimalarial properties [29]. The second set is composed of drugs that directly impair the parasite mitochondrial functions and includes atovaquone, the triazolopyrimidine GW648495 (DSM1 in [30]) and the pyridone GW844520 (Figure S4). GW648495 is an inhibitor of *P. falciparum* dihydroorotate dehydrogenase (DHODH) an essential enzyme of pyrimidine biosynthesis and GW844520, similarly to atovaquone, has been shown to specifically inhibit the cytochrome *bc*1 complex, and to ultimately impair pyrimidine biosynthesis [30]–[32]. We determined the *P. falciparum* killing rate profiles in response to treatments with these drugs. Parasites treated with atovaquone, GW648495, GW844520, and artemisinin were monitored for up to 120 hours. Considering the rapid action of artemisinin, the response to artesunate and artemether was measured at 0, and 24 hours only (Figure 6). The number of viable parasites was decreased by more than 4 log units in all cases, except for GW844520, which showed a slightly delayed parasite reduction profile, as compared to atovaquone and GW648495. This is mostly due to an extension of the lag phase of 24 hours, from 48 to 72 hours, as the log(PRR) values for these three compounds are almost identical, at approximately 3.0 (Table S3). The 99.9% PCTs for three compounds are also very similar, with values ranging from 90 to 108 hours (Table S3). Artemether and artesunate displayed a killing rate apparently similar to artemisinin, with a reduction of 4 log units in parasitemia over 24 hours. (Figure 6). This indicates that compounds inhibiting identical molecular targets or cellular functions, display very similar parasite killing rates, substantiating the idea that drug parasite killing rates are primarily dictated by their antimalarial mode-of-action.

### Single-time point assay allows medium throughput screening for profiling compounds

**Figure 7:**
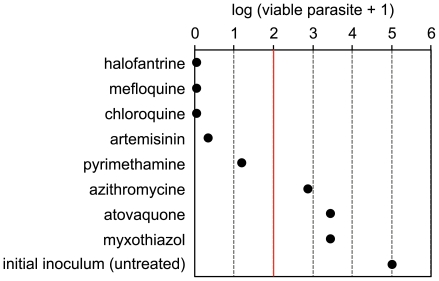
Single time-point viability measurement. *P. falciparum* viability after 72 hours of treatment with the indicated drugs, reported as the log of viable parasite+1, as compared to the untreated controls. The red line represents the threshold of 99.9% parasite reduction. The data are representative of at least 3 independent experiments.

The results presented above indicate that measuring the killing rate profile of candidate antimalarials is a valid approach to identify fast acting compounds. Since parasite viability measurement in a time-course manner and by serial limiting dilution is a relatively labor intensive task, which cannot be fully automatized yet, we explored a single-time point version of the assay, that would allow to screen a larger number of compounds simultaneously. We reasoned that measuring the parasite viability after 72 hours of treatment can be an optimal time point to identify compounds which have either no lag phase or a limited one, and a high *in vitro* PRR value (Figure 5). The effects of 8 compounds, including some already tested in time course experiments, have been analyzed using this single time point approach (Figure 7). Mefloquine, chloroquine, artemisinin, and pyrimethamine induced a reduction in the number of viable parasite higher than 99.9% after 72 hours of treatment, as expected based on the results of initial time-course experiments. Of note, artemisinin value is slightly higher as compared to time-course experiments (0.34 versus 0.13). This is probably due to the fact that drug was not replenished in this single time-point assay. This approach successfully identified atovaquone as a slow acting compound, with a log10 value of 3.4 viable parasites out of 5 from initial inoculum. Halofantrine and azithromycin were identified as fast-, and slow acting drugs, with a reduction in viable parasite down to virtually zero and approximately 3-logs, respectively. Interestingly, myxothiazol, which acts as a *bc*_1_ inhibitor [28], induced a similar reduction in the number of viable parasite as atovaquone, confirming that killing rates correlates with antimalarial mode-of-action. These data indicate that parasite viability measurement at a single-time point of 72 hours is a suitable approach to rapidly identify fast-acting compounds among a large set of candidates.

## Discussion

We present here a comprehensive methodology to measure the net effect of antimalarial compounds on asexual intraerythrocytic *P. falciparum* viability. Although other approaches assessing viability of treated parasites have already been reported [26], [33], those have not studied full time courses for a broad range of compounds, preventing determination of the killing rate profile for the drug. Additionally, in some cases, determination of viable parasites are inferred from the time needed to observe growth. This can be influenced by culture conditions or by the time needed to recover to a steady growth rate following drug exposure, a well documented phenomenon in the case of antibacterials, known as the “postantibiotic effect” [34]. We present here the characterization of an extended set of drugs using a standardized assay. The parasite killing rate in response to drug treatment is determined by directly measuring the number of parasites surviving drug exposure that is the number of parasites able to resume a productive growth upon removal of the drug and addition of fresh erythrocytes. By repeating this measurement over time, it becomes possible to establish multiple parameters, such as the presence of a lag phase as well as to calculate *in vitro* PRR and PCT values. Importantly, any laboratory strain, including adapted *ex vivo* isolates, could potentially be used with the protocol described here. This could be particularly relevant for tolerance phenotypes, which do not translate into altered IC_50_ value, as presently seen in the case of artesunate [4]. The major advantage of the protocol presented here is to directly measure parasite viability and to not rely on parasite metabolic activity or accumulation of specific molecules. This allows to measure compound killing rates regardless of their modes-of-action, which is not always the case with indirect methods [18]. A second advantage is the ability to directly compare compound killing rates by using treatment concentrations based on the compound-specific IC_50_. Direct measurement of viability is more time- and labor-consuming but permits to avoid potential bias or artefacts that appear when viability and parasite metabolic activity become uncoupled. One recently documented example is the dormancy response induced by DHA treatment, which might possibly explain the frequent treatment failures observed with artemisinin treatments [35], [36]. More than 1% of *P. falciparum* treated *in vitro* with a single-dose of DHA were found to be able to recover from a dormancy stage lasting up to 20 days. Parasites becoming dormant in response to DHA, and possibly other antimalarial drugs, are likely to be correctly identified as viable if monitored for re-growth for up to 28 days, which might not be the case using metabolism as surrogate of parasite viability. We also report here a clear case of disconnection between viability and metabolism in response to atovaquone and azithromycin treatments. While more than 90% of parasites are committed to death after 48 hours of treatment with azithromycin, their metabolism stays unaffected up to that time and does not allow to detect this fact. Conversely, atovaquone treated parasites appear to shut down completely their metabolism while a large fraction of the treated parasites is able to survive upon removal of the drug. Altogether this shows that metabolism-based assays do not always fully reflect the killing effects of drugs on parasites and that direct parasite viability monitoring is better suited to detect them.

The analysis of an extended set of antimalarial drugs allowed us to identify different killing rate profiles. The fastest drug tested is artemisinin with an immediate onset of action, an apparent *in vitro* PRR higher than 8.0, and a 99.9% PCT inferior to less than 24 hours. At the other extreme is atovaquone, which displayed a 48 hours lag phase and a low log(PRR) of 2.9, resulting in an overall 99.9% PCT equal to 90 hours. All the other tested drugs fell within this relatively large range of values. It is important to point out that although experiments are performed with unsynchronized parasites, treated cultures are enriched in ring forms (ca. 80%) and this would provoke an extended lag phase on those compounds acting late in the life cycle. The data reported here are in agreement with the published literature, as artemisinin is known to be a fast-acting compound and atovaquone a slow one. For example, clinical PCTs, as determined by microscopy, were reported to be less than 24 hours and more than 60 hours, for artemisinin and atovaquone, respectively [25], [37]. *In vitro* and *in vivo* PRR, derived from clinical data, are measuring different phenomena, *in vitro* PRR measures directly parasite viability while *in vivo* PRR is measuring clearance of the parasites by the organism. *In vivo* artesunate log(PRR) ranges from 2.9 to 3.8 and is lower than the value reported here, estimated at 8.0 [38]–[40]. Similarly, the average PRR for chloroquine observed in clinical studies is lower than the one reported here at, 2.2 and 4.0, respectively [41]–[43]. Available *in vivo* PRR values are all generally lower than the ones measured here *in vitro*, but their ranking is globally conserved between the *in vitro* and *in vivo* situations, suggesting a reasonable correlation between these measures (Figure S5) [37]–[44]. This shift might be due to the fact that *in vivo* PRR are established by comparing the parasitemia before initiating the treatment and 48 hours later without taking lag phase into account or measuring parasite viability. Other intrinsic differences between the *in vivo* physiological situation and the simpler *in vitro* settings, such as drug exposure or parasites sequestration, might also contribute to explain these differences. Regardless, this suggests that *in vitro* PRR is, to some extent, indicative of the therapeutic effect observed in the clinical situation and should therefore be considered as a valuable assay for drug development.

Interestingly, treatments with drug concentrations corresponding to 10 times the IC_50_ appear to be generally sufficient to reach the maximum *in vitro* killing rate, regardless of the mode-of-action of the compounds investigated. This observation might contribute to define therapeutically optimal dosing regimens for compounds entering the clinical candidate stage and might facilitate their characterization at relevant concentrations in efficacy trials. The investigation of several drugs sharing the same pharmacophore, such as artemisinin, artesunate, and artemether or targeting the same parasite organelle, in this case the mitochondrion, such as atovaquone, GW648495, and GW8445520, indicated that similar viability profiles were observed among these set of compounds. This suggests that the protocol presented here is a valuable tool to investigate compounds with an antimalarial activity of unknown mode-of-action, as possible mode-of-action could be excluded or envisaged based on comparison with available data (although additional information on the compound should be needed to assign a definitive mode of action) This approach would also allow to identify potential new mode-of-action, possibly able to overcome established resistance to current antimalarials.

Speed-of-action is one of the main determinant of antimalarial compound efficacy and is a crucial clinical parameter, which will determine not only the *in vivo* PCT but also the time needed to clear patient symptoms, which should ideally be as fast as possible. Another important aspect related to drug speed-of-action is the resistance opportunity window that is occurring when a drug is facing parasites in a clinical situation, that is the time window during which the parasite are exposed to suboptimal drug-level that might drive selection and transmission of resistant parasites. It is argued that fast-acting antimalarial will minimize the chance of resistant mutants to arise within a patient and should be favored. The *in vitro* methodology presented here allows to rapidly and easily identify fast-acting compounds, which present added value for clinical development. Importantly, speed-of-action and PCT do not depend on drug potency, prompting the need for a standardized *in* vitro method to investigate these factors. The methodology presented here addresses this issue. We also identified that 72 hours of treatment is an optimal time-point to accurately discriminate between fast- and slow-acting compounds, although other endpoints could also be used. In fact, compounds with a very slow rate of killing, like GW844520 (Figure 6), would hardly show an effect at 72 hours and 96 hours treatment should be more appropriated to detect effects by this kind of compounds. This streamlined version allows to test more compounds simultaneously and might be the first step toward establishing a higher-throughput assay. Such an assay would prove to be very useful to screen the Tres Cantos Antimalarial Compound set (TCAMS), as well as other large library of active antimalarial compounds [45]–[47]. This would facilitate the identification of compounds not only potent but also fast acting against *P. falciparum* parasites.

Altogether, we present a standardized assay to measure the net effect of compound on parasite viability, allowing to characterize a crucial parameter that is the killing rate. We provide data for an extended set of classical antimalarials which will serve as benchmark references for future studies and hopefully the discovery of compounds as rapid as artemisinin derivatives, which would have the potential to replace them, if needed, and be the next generation of fast acting antimalarials that we could oppose to the parasite.

## Materials and Methods

### Parasite clones and culture conditions

The *P. falciparum* strain 3D7A used in this study was obtained from the Malaria Research and Reference Reagent Resource Center (MR4). Accurate description can be obtained at http://www.mr4.org. Red blood cells were obtained from the Spanish Red Cross Blood Bank. *P. falciparum* were cultured using standard procedures as described previously [48]. An inoculum of parasitized red blood cells (PRBC) at 0.5% parasitemia and 2% hematocrit in RPMI-1640 , 5% AlbuMAX, 2% D-sucrose, 0.3% L- glutamine and 150 µM hypoxanthine was used for the assays described below, if not otherwise stated.

### Hypoxanthine incorporation assay

Intraerythrocytic *P. falciparum* growth inhibition by drugs was determined using a modification of the *in vitro* [^3^H]-hypoxanthine incorporation method [9]. Briefly, asynchronous cultures (ca. 80% rings) of PRBC at 2% hematocrit and 0.5% parasitemia with 5 µM hypoxanthine were exposed to 2-fold serial dilutions of the compounds (Table S4). 96 well plates (Costar #3894) were incubated 24 h at 37°C, 5% CO_2_, 5%O_2_, 95% N_2_. After 24 h of incubation, [^3^H]-hypoxanthine was added and plates were incubated for an additional 24 hours. After that period, plates were harvested on glass fiber filters (Wallac #1450-421) using a cell harvester 96 (TOMTEC, Perkin Elmer). Filters were dried and melt-on scintillator sheets (MeltiLex A, PerkinElmer #1450-441) used to determine the incorporation of [^3^H]-hypoxanthine . Radioactivity was measured using a microbeta counter (Perkin Elmer). Data are normalized using the incorporation of the positive control, (PRBC without drug). IC_50_ values were determined using Excel and Grafit 5 software. The assays were performed in at least three independent experiments, and standard deviations have been calculated.

### *In vitro* parasite viability assay

PRBC, under the conditions mentioned above (asynchronous cultures with a predominant ring population), are treated with the selected drugs at concentration corresponding to multiple of their respective IC_50_ (Table S1). Drug is renewed daily over the entire treatment period. Samples of untreated parasite (0 h) and of treated parasites (24 h, 48 h, 72 h, 96 h and 120 h time-points) are aliquoted to perform serial dilutions in 96 well plates (Costar #3894) by adding fresh erythrocytes and new culture media. Previously to the serial dilutions the drug is washed out using fresh culture media.

Parasites are cultured for up to 28 days to allow wells with viable parasites to render detectable parasitemia. Samples are taken to examine growth using [^3^H]-hypoxanthine incorporation (see above, although any alternative method to detect parasitemia can be used) at days 21 and 28. The number of viable parasites is back-calculated by using the formula X^n−1^ where n is the number of wells able to render growth and X the dilution factor (when n = 0 number of viable parasites is estimated as zero). Briefly, 0.1 ml of drug-free culture (approximately 10^5^ parasites) is dispensed in the first well of a 96 well microtiter plate. For 2-fold serial dilutions 50 µl of culture containing parasites is transferred to the next well containing 50 µl of fresh erythrocytes at 2% hematocrit. For each time point 20 serial dilutions of at least four replicates are performed using two different 96 well plates for parasite culture. This allows to count up to 5×10^5^ viable parasites per time point. For 3-fold serial dilutions 50 µl of culture is transferred to the next well containing 100 µl of fresh erythrocytes at 2% hematocrit. For each time point 12 serial dilutions of at least four replicates are performed using two different 96 well plates for parasite culture. This allows to count up to 2×10^5^ viable parasites per time point. For 10-fold serial dilutions 10 µl of culture is transferred to the next well containing 90 µl of fresh erythrocytes at 2% hematocrit. For each time point 8 serial dilutions of at least four replicates are performed. This allows to count up to 10^7^ viable parasites per time point. Accuracy of the method is presented in the Results section in the main text and represented in Figure S1. Lag phase, log(PRR) and PCT values are calculated as described in the main text.

## Supporting Information

Figure S1 **Validation of parasite viability determination by serial dilution.** **A**. A population of 10^6^ parasites/ml, as determined by microscopy, at 0.5% parasitemia and 2% hematocrit was used to perform 1/2, 1/3, and 1/10 serial limiting dilutions on aliquots of 10^5^ parasites. Parasite growth was measured after 21 days and the highest dilution displaying growth, as determined by hypoxanthine incorporation, was considered to back calculate the initial number of parasite. **B**. Number of parasites calculated based on different dilution factors. Error bars are standard deviations of at least 8 repetitions from one experiment. The values are representative of three independent experiments.
(EPS)

Figure S2 **Effect of media change for unstable drugs.** *P. falciparum* viability time-course profiles in response to artemisinin treatment performed with and without media exchange and drug replenishment every 24 hours. A few parasites (<3) are still viable after 48 h treatment without replenishment of the drug.
(EPS)

Figure S3 **Parasite viability in response to different concentrations of drugs.** *P. falciparum* viability time-course profiles in response to chloroquine and lumefantrine treatments at concentrations corresponding to 1×, 3×, 10×, and 100× their respective IC_50_. Error bars are SEM of at least 4 independent experiments.
(EPS)

Figure S4 **Chemical structures of GW648495 and GW844520.**
(EPS)

Figure S5 **Comparison between** ***in vitro*** **and** ***in vivo*** **PRR data.** *In vivo* PRR data have been estimated from available clinical trial data and are compared to the *in vitro* PRR values determined in this study. Drug ranking is generally conserved between the *in vitro* and *in vivo* situations, with the exception of pyrimethamine. Of note, pyrimethamine *in* vivo value is derived from data obtained in combination with sulfadoxine. Artemether (arte), lumefantrine (lum), chloroquine (chq), pyrimethamine (pyri), mefloquine (mef), azithromycin (azi). *In vivo* PPR value for artemether and lumefanterine were calculated from clinical trial data available on the Food and Drug Administration website (http://www.fda.gov/ohrms/dockets/ac/08/briefing/2008-4388b1-02-Novartis.pdf).
(EPS)

Table S1 **50% inhibitory concentration (IC_50_) of drugs used in this study as determined by radio-labeled hypoxanthine incorporation.**
(DOC)

Table S2 ***In vitro*** **parasite reduction ratio and clearance time in response to various concentrations of chloroquine and lumefantrine.**
(DOC)

Table S3 ***In vitro*** **parasite reduction ratio and clearance time in response to atovaquone, GW648495X, GW844520X.**
(DOC)

Table S4 **Provider and reference number of the compounds used in this study.**
(DOC)
