# Supplementary material for: P. falciparum In Vitro Killing Rates Allow to Discriminate between Different Antimalarial Mode-of-Action
Source: PLoS One. 2012 Feb 23;7(2):e30949. doi: 10.1371/journal.pone.0030949 (PMC3285618; doi:10.1371/journal.pone.0030949)
Supplement: Table S1 — 50% inhibitory concentration (IC50) of drugs used in this study as determined by radio-labeled hypoxanthine incorporation. (DOC) [file pone.0030949.s006.doc]

**Table S1.**

|  | IC_50_ (µM) ^a^ | treatment time (h) |
| --- | --- | --- |
| artemether | 0.011±0.005 | 48 |
| artemisinin | 0.032±0.015 | 48 |
| artesunate | 0.029 ±0.002 | 48 |
| atovaquone | 0.001±0.0004 | 48 |
| azithromycin | 0.272±0.11 | 96 |
| chloroquine | 0.024± 0.008 | 48 |
| GW648495X | 0.136±0.010 | 48 |
| GW844520X | 0.050±0.004 | 48 |
| halofantrine | 0.0019±0.003 | 48 |
| lumefantrine | 0.009±0.002 | 48 |
| mefloquine | 0.043±0.014 | 48 |
| myxothiazol | 0.018±0.007 | 48 |
| piperaquine | 0.021±0.003 | 48 |
| pyrimethamine | 0.094±0.029 | 48 |
| pyronaridine | 0.005±0.001 | 48 |

^a^ average and standard deviation from at least 12 measurements in two independent experiments.
