# Supplementary material for: P. falciparum In Vitro Killing Rates Allow to Discriminate between Different Antimalarial Mode-of-Action
Source: PLoS One. 2012 Feb 23;7(2):e30949. doi: 10.1371/journal.pone.0030949 (PMC3285618; doi:10.1371/journal.pone.0030949)
Supplement: Table S2 — In vitro parasite reduction ratio and clearance time in response to various concentrations of chloroquine and lumefantrine. (DOC) [file pone.0030949.s007.doc]

**Table S2.**

|  | chloroquine | | lumefantrine | |
| --- | --- | --- | --- | --- |
|  | log(PRR) | 99.9% PCT (h) | log(PRR) | 99.9% PCT (h) |
| 1x IC_50_ | 3.0 | 48 | 4.1 | 43 |
| 3x IC_50_ | 4.3 | 33 | 4.7 | 33 |
| 10x IC_50_ | 4.5 | 32 | 4.8 | 32 |
| 100x IC_50_ | 4.2 | 36 | 4.8 | 30 |
