# Supplementary material for: P. falciparum In Vitro Killing Rates Allow to Discriminate between Different Antimalarial Mode-of-Action
Source: PLoS One. 2012 Feb 23;7(2):e30949. doi: 10.1371/journal.pone.0030949 (PMC3285618; doi:10.1371/journal.pone.0030949)
Supplement: Table S3 — In vitro parasite reduction ratio and clearance time in response to atovaquone, GW648495X, GW844520X. (DOC) [file pone.0030949.s008.doc]

**Table S3.**

|  | lag phase (h) | log(PRR) | 99.9% PCT (h) |
| --- | --- | --- | --- |
| atovaquone | 48 | 2.9 | 90 |
| GW648495X | 48 | 3.4 | 90 |
| GW844520X | 72 | 3.0 | 108 |
