# Supplementary material for: P. falciparum In Vitro Killing Rates Allow to Discriminate between Different Antimalarial Mode-of-Action
Source: PLoS One. 2012 Feb 23;7(2):e30949. doi: 10.1371/journal.pone.0030949 (PMC3285618; doi:10.1371/journal.pone.0030949)
Supplement: Table S4 — Provider and reference number of the compounds used in this study. (DOC) [file pone.0030949.s009.doc]

**Table S4.**

| Compound | Provider | Catalog number |
| --- | --- | --- |
| artemether | Sigma | A9361 |
| artemisinin | Sigma | 36159-3 |
| artesunate | Sigma | A3731 |
| atovaquone | GlaxoSmithkline |  |
| azithromycin | Apin Chemicals LTD | 26198A |
| chloroquine | Sigma | C6628 |
| GW648495 | GlaxoSmithkline |  |
| GW844520 | GlaxoSmithkline |  |
| halofantrine | Sigma | H9414 |
| lumefantrine | MMV000014 (PCMMVJB-0014) |  |
| mefloquine (racemic) | MMV000015 (PCMMVJB-0015) |  |
| myxothiazol | Sigma | M-5779 |
| piperaquine | MMV000022 (PCMMVJB-0022) |  |
| pyrimethamine | Sigma | P-7771 |
| pyronaridine | MMV000025 (PCMMVJB-0025) |  |
